# Pharmacological inhibition of Bmi1 by PTC-209 impaired tumor growth in head neck squamous cell carcinoma

**DOI:** 10.1186/s12935-017-0481-z

**Published:** 2017-11-21

**Authors:** Qiong Wang, Zhongwu Li, Yaping Wu, Rong Huang, Yumin Zhu, Wei Zhang, Yanling Wang, Jie Cheng

**Affiliations:** 1 Jiangsu Key Laboratory of Oral Disease, Nanjing Medical University, 136 Hanzhong Road, Nanjing, 210029 Jiangsu China; 2 Department of Oral and Maxillofacial Surgery, Affiliated Stomatological Hospital, Nanjing Medical University, 136 Hanzhong Road, Nanjing, 210029 Jiangsu China; 3 Department of Oral Pathology, School of Stomatology, Nanjing Medical University, 136 Hanzhong Road, Nanjing, 210029 Jiangsu China

**Keywords:** Head neck squamous cell carcinoma, Polycomb, Bmi1, PTC-209

## Abstract

**Background:**

Bmi1 (B lymphoma Mo-MLV insertion region 1 homolog) contributes to human tumorigenesis via epigenetic transcriptional silencing and represents a novel therapeutic target with great potentials. Here we sought to determine the therapeutic efficiency of PTC-209, a potent and selective Bmi1 inhibitor, in head neck squamous cell carcinoma (HNSCC) cells and a HNSCC xenograft model.

**Methods:**

The mutation pattern, mRNA level of Bmi1 in HNSCC and its associations with clinicopathological parameters were determined through comprehensive data mining and interrogation using publicly available databases GENT, cBioPortal, Oncomine and TCGA. The PTC-209, a selective and potent Bmi1 inhibitor, was exploited and its effect on Bmi1 expression was measured in two HNSCC cell lines Cal27 and FaDu. The phenotypical changes of HNSCC cells were observed upon PTC-209 treatment in vitro. Moreover, the therapeutic effects of PTC-209 for HNSCC were determined in a xenograft animal model.

**Results:**

Through comprehensive data mining and interrogation, we found that Bmi1 mRNA was frequently overexpressed in a subset of HNSCC samples. Our data revealed that PTC-209 robustly reduced the expression of Bmi1 in Cal27 and FaDu cells presumably by post-transcriptional repression and ubiquitin-proteasomal degradation. PTC-209 treatment resulted in impaired cell proliferation, G1-phase cell cycle arrest, compromised migration and invasiveness, and increased cell apoptosis and chemosensitivity to 5-FU and cisplatin in vitro. Moreover, PTC-209 exposure reduced colony formation, tumorsphere formation and the percentage of ALDH1^+^ subpopulation in both Cal27 and FaDu cells. Importantly, in vivo PTC-209 administration significantly reduced tumor growth in a HNSCC xenograft model probably by Bmi1 inhibition and impaired cell proliferation.

**Conclusions:**

Our findings indicate that pharmacological inhibition of Bmi1 is a novel therapeutic strategy for HNSCC patients, especially with those with aberrant Bmi1 overexpression.

**Electronic supplementary material:**

The online version of this article (10.1186/s12935-017-0481-z) contains supplementary material, which is available to authorized users.

## Background

Head neck squamous cell carcinoma (HNSCC) is the sixth most common cancers and one of the leading cancer-related death worldwide. The major etiological risks for this malignancy include smoking abuse, alcohol consumption, betel quid chew and human papillomavirus (HPV) infection [1]. Despite tremendous advancement in multimodal therapies against HNSCC over the past decades, however, the long-term survival rate for these devastating diseases, especially for patients with advanced diseases, has not been markedly improved [2]. Locoregional relapse, cervical lymph node metastasis and therapeutic resistance are recognized as the prevalent factors affecting patient prognosis. Although intensive efforts have been made to unravel the genetic and environmental factors driving HNSCC tumorigenesis, however, mechanistic understanding about HNSCC tumorigenesis still remains far from complete [3, 4]. Therefore, identification and verification of new biomarkers and therapeutic targets for HNSCC are paramount and urgent to improve treatment outcome [5].

Polycomb group proteins are key chromatin modulators governing cell fate decision, stem cell self-renewal and differentiation, and tumorigenesis largely via transcriptional repression of a plethora of downstream targets [6]. B lymphoma Mo-MLV insertion region 1 homolog (Bmi1) is a core member of the Polycomb Repressive Complex 1 (PRC1) required for monoubiquitination of histone 2A, usually functions as an epigenetic silencer of target genes such as Ink4a-arf locus [6, 7]. Accumulating evidence has been indicated that Bmi1 is critically involved in diverse fundamental cellular processes such as cell proliferation, apoptosis, senescence, epithelial–mesenchymal transition (EMT) and stem cell maintenance [7–9]. Aberrant overexpression of Bmi1 has been found in multiple human malignancies such as lung, liver, breast and head neck cancer. Its overexpression often correlates with advanced stages, aggressive clinicopathological behaviors, therapeutic resistance and unfavorable prognosis in these cancers [9–12]. Enforced overexpression of Bmi1 facilitated malignant transformation, cancer cell proliferation, EMT and metastatic spreading, whereas its depletion inhibited cell proliferation, migration and invasion and induced cell apoptosis and senescence both in vitro and in vivo [9, 13–15]. Noticeably, Bmi1 plays critical and indispensable roles in governing self-renewal capacity of normal and malignant cancer stem cells (CSCs) which the latter has been increasingly recognized to largely responsible for cancer initiation, therapeutic resistance, disease relapse and metastatic dissemination [8, 16–18]. Several target genes including p16 and E-cadherin have been identified to be responsible for these essential oncogenic roles of Bmi1 in diverse cancer contexts including HNSCC [7, 9]. These abovementioned findings demonstrate that Bmi1 is not only an oncogenic driver during tumorigenesis, but also represents an cancer biomarker and therapeutic target with translational significance. Indeed, genetic silencing and pharmacological inhibition of Bmi1 induced apoptosis and senescence, enhanced chemosensitivity, and diminished invasive and metastatic potentials, thus ultimately compromised cancer progression [9, 19, 20]. Particularly, targeting Bmi1 with a selective small-molecule inhibitor (PTC-209) resulted in loss of colorectal CSCs and caused long-term, irreversible impairment of tumor progression [12, 21, 22]. Thus, these findings support the notion that chemical targeting Bmi1 might be an attractive and plausible way to eradicate cancers as a novel therapeutic strategy [23].

Several lines of evidence have revealed that Bmi1 associates with malignant transformation of precancerous lesions, EMT and CSCs maintenance of HNSCC [9, 13, 24]. Elevated Bmi1 usually correlated with aggressive features and unfavorable patients’ survival [11, 13, 20, 25]. Our previous study has found that Bmi1 was aberrantly overexpressed in oral tongue cancer and it was potently inhibited by HDACi (inhibitors of histone deacetylases) chemicals, which in turn resulted in impaired tumor growth. However, these chemicals are not specific and might induced unwanted downstream effects beyond Bmi1 inhibition [20]. In the present study, we sought to determine the therapeutic efficiency of PTC-209, a novel and selective inhibitor of Bmi1, against HNSCC using both in vitro cell culture and in vivo xenograft animal model.

## Materials and methods

### HNSCC cell lines and chemical reagents

Two HNSCC cell lines Cal27 and FaDu were purchased from American Type Culture Collection (ATCC) and authenticated by short tandem repeat (STR) profiling and tested for mycoplasma. Cells were grown in DMEM/F12 (Invitrogen) supplemented with 10% FBS (Gibco) and penicillin–streptomycin (1%), and maintained at 37 °C in a 5% CO_2_-humidified incubator. PTC-209 was purchased from Selleck (catalog No. S7372) and dissolved in DMSO. Two common anticancer agents 5-fluorouracil (5-FU) and cisplatin were purchased from Sigma-Aldrich, dissolved as stocking solutions and diluted with culture medium upon additions to cell plates. HNSCC cells were treated with diverse concentrations of PTC-209 alone or in combination with 5-FU or cisplatin for indicated times and then harvested for further analyses.

### RNA extraction and real-time RT-PCR

Total RNA was extracted from cells using Trizol reagent (Invitrogen) and then reversely transcribed into first strand cDNA using PrimeScript™ RT reagent kit (Takara). Then the cDNA was subjected to real-time PCR reaction using SYBR Premix Ex Taq™ kit (Takara) following the manufacturer’s instructions. The gene-specific primers for human Bmi1, Ezh2, p16 and GAPDH were purchased commercially (Invitrogen). Relative mRNA expression of each gene as compared to internal control GAPDH was quantified using comparative CT method.

### Western blot and immunoprecipitation assay

Cells in culture flasks or plates were lysed in ice-cold buffer containing protease inhibitor cocktail (Roche). Equal amounts of protein samples were loaded and separated by 8–12% SDS-PAGE and transferred to PVDF membranes (Millipore) followed by 5% non-fat milk or 3% BSA blocking. These blots were incubated at 4 °C overnight with primary antibodies against Bmi1, p16, cleaved-PARP, Sox2, Nanog, Oct4 (Cell signaling, 1:1000 dilution), E-cadherin, N-cadherin and Vimentin (BD Biosciences, 1:2000 dilution), Lin28B (Abcam, 1:1000 dilution), ubiquitinated histone 2A and total histone 2A (Millipore, 1:1000 dilution), and GAPDH (Santa Cruz, 1:1000 dilution) followed by incubations with the corresponding secondary antibodies. The relative levels of each protein were quantified with Quantity One software (Bio-Rad). The protein–protein interaction was determined by protein immunoprecipitation using Pierce™Co-Immunoprecipitation Kit (ThermoFisher) and performed according to the manufacturer’s protocol. The antibodies for IP and following western blot were anti-Bmi1 (Cell signaling, #6964) and anti-ubiquitin (Cell signaling, #3933, 1:1000).

### MTT assay

Cell proliferation and viability were determined by absorbance using MTT assay. Approximately 1000–3000 cells per well were seeded in the 96-well plates. At the indicated time-points, 5 mg/ml MTT (Sigma) was added to the plates and incubated at 37 °C for another 4 h. Absorbance at 490 nm was measured with an automatic enzyme-linked immunosorbent assay reader (BioTek Instruments).

### Flow-cytometric analysis and ALDEFLUOR assay

For cell apoptosis assay, cells were stained with Annexin V: PI Apoptosis Detection Kit (BD Bioscience). The ALDEFLUOR assay was used to monitor the percentage of ALDH1^+^ cells in HNSCC treated with or without PTC-209 with a FACSCalibur flow cytometer. A specific ALDH1 inhibitor, diethylaminobenzaldehyde, was used as a negative control.

### In vitro cell migration and wound healing assay

Cell invasion assay was performed using chambers (8-μm pore size, Corning) in 24-well plates. Forty-eight hours after PTC-209 treatment, cells were detached and resuspended, then seeded into the upper chambers with medium containing 1% FBS. Complete medium containing 10% FBS in the lower chambers served as chemoattractant. The non-invading cells were gently removed with a cotton swab and those migratory cells located on the lower side were stained with crystal violet. The number of migrated cells were counted and averaged in randomly selected 10 fields under microscope. For wound healing assay, cells were grown into confluent monolayers and scratched using a sterile 200 µl pipette. Cell migration was observed at various time-points later by microscopy. Images of 10 scratches per cells were captured during the experiment and compared with Image J software.

### Colony formation and tumorsphere formation assays

For colony formation assay, 1000 single cells pretreated with PTC-209 or vehicle were seeded into 6-well plates or dishes and allowed to grow for 10–14 days. The cells were then fixed and stained with crystal violet. The colonies were further visualized under an invert microscope and photographed. Cell aggregations with more than 50 cells were defined as colonies and counted.

For tumorsphere assay, single cells pretreated with PTC-209 or vehicle were seeded at a density of 1000/well in ultra-low attachment plates and cultured in serum-free DMEM/F12 supplemented with 1% B27 and N2 supplements as well as human recombinant epidermal growth factor (EGF, 20 ng/ml, R&D systems) and basic fibroblast growth factor (bFGF, 10 ng/ml, R&D systems). The fresh media were changed every other day until the tumorsphere formed. For passaging, the tumorspheres were collected by centrifugation and dissociated by 0.5% trypsin to obtain single cells which were further cultured for secondary tumorsphere formation. The tumorsphere with a diameter over 100 μm were counted and recorded 2 weeks after plating.

### PTC-209 treatment in HNSCC xenograft model

All the animal protocols (2016-168) were in accordance with institutional animal welfare guidelines of Nanjing Medical University. Both 2 × 10^6^ Cal27 and FaDu cells in 100 μl PBS were injected subcutaneously into nude mice (male, aged 8 weeks). Three weeks later, these mice bearing tumors (approximate 100 mm^3^ in volume) were randomly divided into two groups (6 mice per group) which were scheduled to receive the following treatments: 30 mg/kg PTC-209 every day by subcutaneous injection or vehicle (PBS) only in control animals for consecutive 15 days. The tumor diameters were measured by calipers every 3 days. Tumor volume was calculated by the formula volume (mm^3^) = [length (mm) × width (mm)^2^] × 0.5. Tumor weight was also measured upon tumor samples were harvested.

Immunohistochemical staining for Bmi1 and Ki-67 in these samples was performed and scored similarly as our previous reports [26, 27]. The immunoreactivity in each slide was assessed independently by two senior oral pathologists without knowledge about the clinical and pathological information. Negative controls (without primary antibody incubation) were included in each staining run. Immunoreactivity was semi-quantitatively evaluated on the basis of staining intensity and distribution using the immunoreactive score. Immunoflurescent staining of CD44 (ab51037, Abcam) in samples were also conducted as we previously documented. The images were further visualized under fluorescence microscope.

### Data mining and analysis of Bmi1 in HNSCC via publicly available database

The original data concerning mutational landscape and expression of Bmi1 mRNA in HNSCC were retrieved from three publicly available databases including GENT (http://medical-genome.kribb.re.kr/GENT/) [28], cBioPortal (http://www.cbioportal.org/) [29], Oncomine (https://www.oncomine.org/) [30] and TCGA (https://cancergenome.nih.gov/). The expression levels of Bmi1 mRNA (log2-transformed) in HNSCC and normal counterparts were retrieved and statistically compared. The associations between expression status of Bmi1 (high or low using median value as cutoff) and clinicopathological parameters as well as overall survival were determined by Chi square test or Kaplan–Meir analysis (Log-rank test), respectively.

### Statistical analysis

All quantitative data in the present study was shown as mean ± SD of two or three independent experiments and compared with Student’s *t* test or ANOVA with Bonferroni post hoc test unless otherwise specified. *p* values less than 0.05 (two-sided) were considered statistically significant. All statistical analyses were performed using Graphpad Prism 6 or SPSS 18.0 software.

## Results

### Bmi1 mRNA is overexpressed in a subset of HNSCC samples

**Fig. 1:**
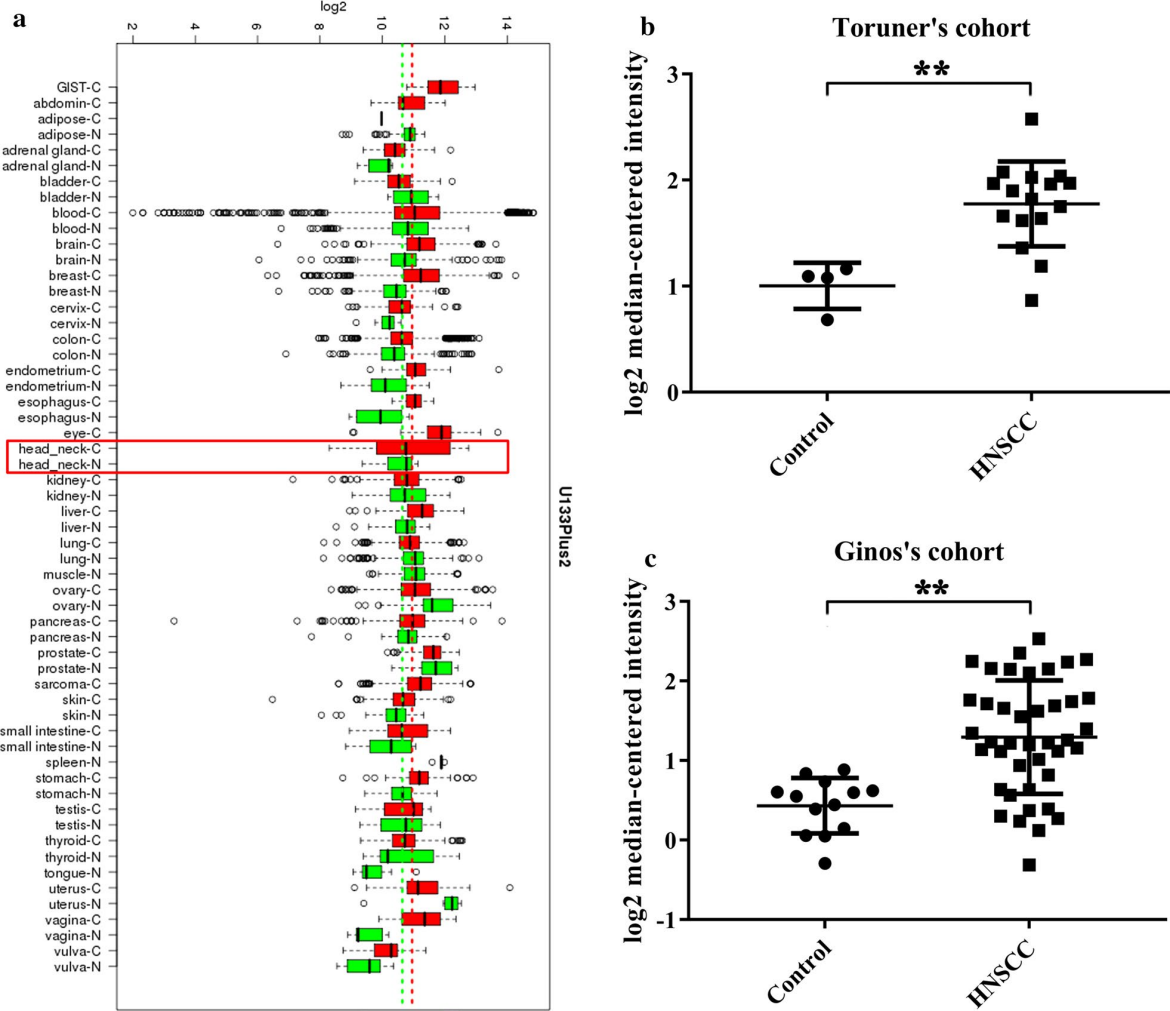
Overexpression of Bmi1 mRNA in a subset of HNSCC. **a** Transcriptional landscape of Bmi1 in a broad spectrum of human cancers as compared to the corresponding normal tissue was revealed using GENT database consisting of the cancer profiling data using U133Plus2 platform. X-axis represents the pairs of cancer and corresponding normal counterparts. Y-axis represents the log2 median–centered intensity. The mRNA levels of Bmi1 were compared between HNSCC samples and normal counterparts in Toruner’s (**b**) and Ginos’s (**c**) patient cohorts. The original data were retrieved from Oncomine database and plotted using Graphpad Prism 6.0 software. Y-axis represents the median intensity, 25th and 75th percentile data. **p < 0.01, Student-*t* test for Toruner’s data and Mann–Whitney U test for Ginos’s data

Our previous studies and others have indicated that Bmi1 is aberrantly overexpressed in oral tongue cancer and HNSCC samples with both diagnostic and prognostic values [9, 15, 20, 25]. To extend these findings and reinforce this notion, we utilized the publicly available databases including GENT, cBioPortal, Oncomine and TCGA to interrogate the mutational landscape and expression pattern of Bmi1 in HNSCC. As shown in Fig. 1a, transcriptional profiling data of human cancer in GENT revealed that the mRNA level of Bmi1 varied remarkably among diverse human cancers and its abundance was frequently higher in cancers than those normal counterparts including HNSCC. Data mining from Oncomine database indicated marked overexpression of Bmi1 mRNA in HNSCC samples from Toruner’s [31] and Ginos’s [32] patient cohorts, but comparable in HNSCC samples from Cromer’s [33], Kuriakose’s [34] as well as Peng’s [35] cohorts (Fig. 1b, c and data not shown). Unexpectedly, interrogation of TCGA HNSCC dataset revealed that the abundance of Bmi1 mRNA in HNSCC samples (502 cases) was comparable to normal epithelial (44 cases). No significant associations between Bmi1 expression (median value as cutoff between low and high expression) and aggressive clinicopathological parameters and patients survival were identified (Additional file 1: Figure S1, Additional file 2: Table S1). Further bioinformatics analyses in cBioPortal database revealed that total frequencies of Bmi1 genetic amplification, mutation and deletion in HNSCC samples were less than 3%, suggesting that gene structural variations might not be primarily responsible for its expression pattern in HNSCC. Taken together, these analyses from bioinformatics data mining and interrogation suggest that Bmi1 mRNA is aberrantly overexpressed in a fraction of HNSCC and might serve as putative oncogene during HNSCC initiation and progression.

### PTC-209 reduces Bmi1 expression probably by inhibiting its transcription and inducing its protein degradation in HNSCC cells

**Fig. 2:**
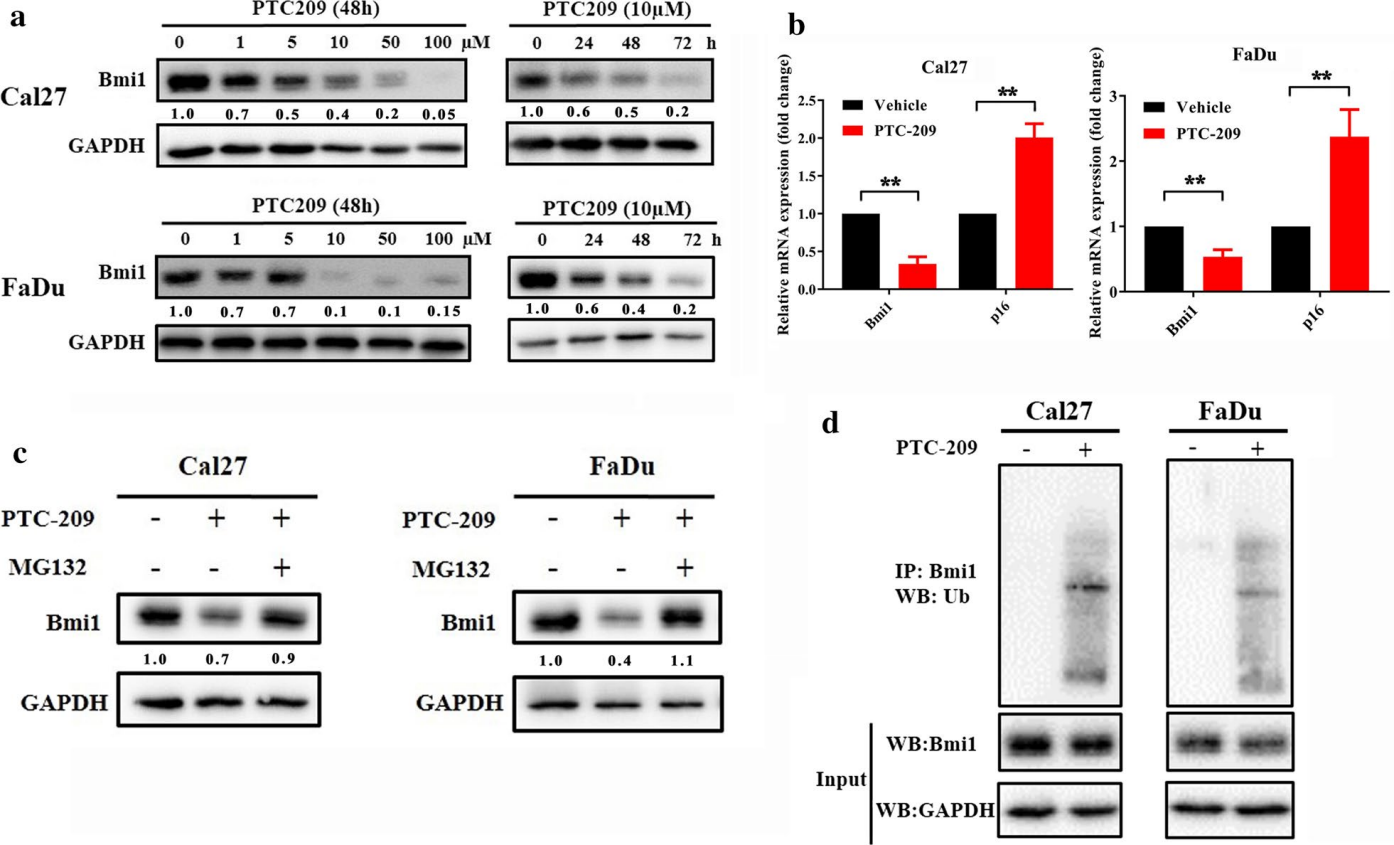
PTC-209 reduced Bmi1 expression by transcriptional repression and protein degradation in HNSCC cells. **a** Endogenous Bmi1 protein was efficiently inhibited by PTC-209 in a time- and dosage-dependent manner in Cal27 and FaDu cells for indicated times. Representative images of WB are shown. **b** The mRNA levels of Bmi1 were significantly decreased following PTC-209 exposure (10 μM) for 48 h, and its well established downstream target p16 was derepressed. **c** The Bmi1 inhibition by PCT-209 treatment (10 μM, 48 h) was partially attenuated by pretreatment with the ubiquitin-proteinase inhibitor MG132 (5 μM, 6 h). Representative images are shown. **d** The ubiquitination of Bmi1 was measured in both cells treated with PTC-209 (10 μM, 24 h) followed by MG-132 (10 μM) for additional 6 h. Total cell lysates were immunoprecipitated with anti-Bmi1 antibody and then blotted for ubiquitin (Ub). GAPDH was used as a loading control. Representative images are shown. Data showed here are mean ± SD from three independent experiments, **p < 0.01, Student’s *t* test

Accumulating evidence has revealed that Bmi1 represents a promising therapeutic target with considerable translational potentials [23]. The pioneering studies have identified PTC-209 as an novel chemical inhibitor of Bmi1 through chemical library compound screen and demonstrated its potency and specificity against Bmi1 in vitro and in vivo [21, 22]. We wondered whether PTC-209 had the similar inhibitory effect on Bmi1 and induced therapeutic effects in HNSCC. To address this issue, we initially selected two HNSCC cell lines Cal27 and FaDu with relatively high endogenous Bmi1 and incubated them with diverse concentrations of PTC-209 and then monitored the expression changes of Bmi1. Following the exposure of PTC-209 for 48 h, the protein abundance of Bmi1 in both cells was significantly reduced as compared to vehicle-treated cells (Fig. 2a, left panel). Moreover, when cells were exposed during a 72 h time course, the Bmi1 protein levels were greatly diminished in a time-dependent manner (Fig. 2a, right panel). To understand the mechanisms underlying Bmi1 reduction upon PTC-209 exposure, the changes of Bmi1 mRNA abundance were measured by real-time RT-PCR. As displayed in Fig. 2b, PTC-209 treatment (10 μM, 48 h) resulted in significant downregulation of Bmi1 transcripts and de-repression of its well-established downstream target p16. In addition, global ubiquitinated histone 2A (uH2A), the hallmark of Bmi1-mediated repressive chromatin structures and transcriptional silencing, was significantly downregulated upon PTC-209 treatment (10 μM, 48 h, Additional file 3: Figure S2). However, the expression of other polycomb members EZH2, SUZ12 and EED was not affected (data not shown), thus partially supporting the highly selective nature of PTC-209 against Bmi1. Moreover, considering the relatively instability of Bmi1 protein [23, 36], we next sought to explore whether PTC-209 affected the turnover of Bmi1 protein in HNSCC cells. To this regard, we monitored the changes of Bmi1 protein when cells were treated with PTC-209 alone or in combination with the proteinase inhibitor MG132. Intriguingly, as shown in Fig. 2c, Bmi1 reduction induced by PTC-209 exposure (10 μM) was partially attenuated by MG132, thus suggesting that PTC-209 might induce Bmi1 downregulation partially by triggering its protein degradation. Furthermore, as shown in Fig. 2d, our data revealed that the ubiquitination of Bmi1 protein was markedly increased following PTC-209 exposure in vitro. Collectively, these data clearly indicate that Bmi1 can be pharmacologically inhibited by PTC-209 in HNSCC and this effect is presumably induced by post-transcriptional repression and protein degradation.

### PTC-209 inhibited cell proliferation, migration and invasion, and induced cell cycle arrest and apoptosis in HNSCC cells

**Fig. 3:**
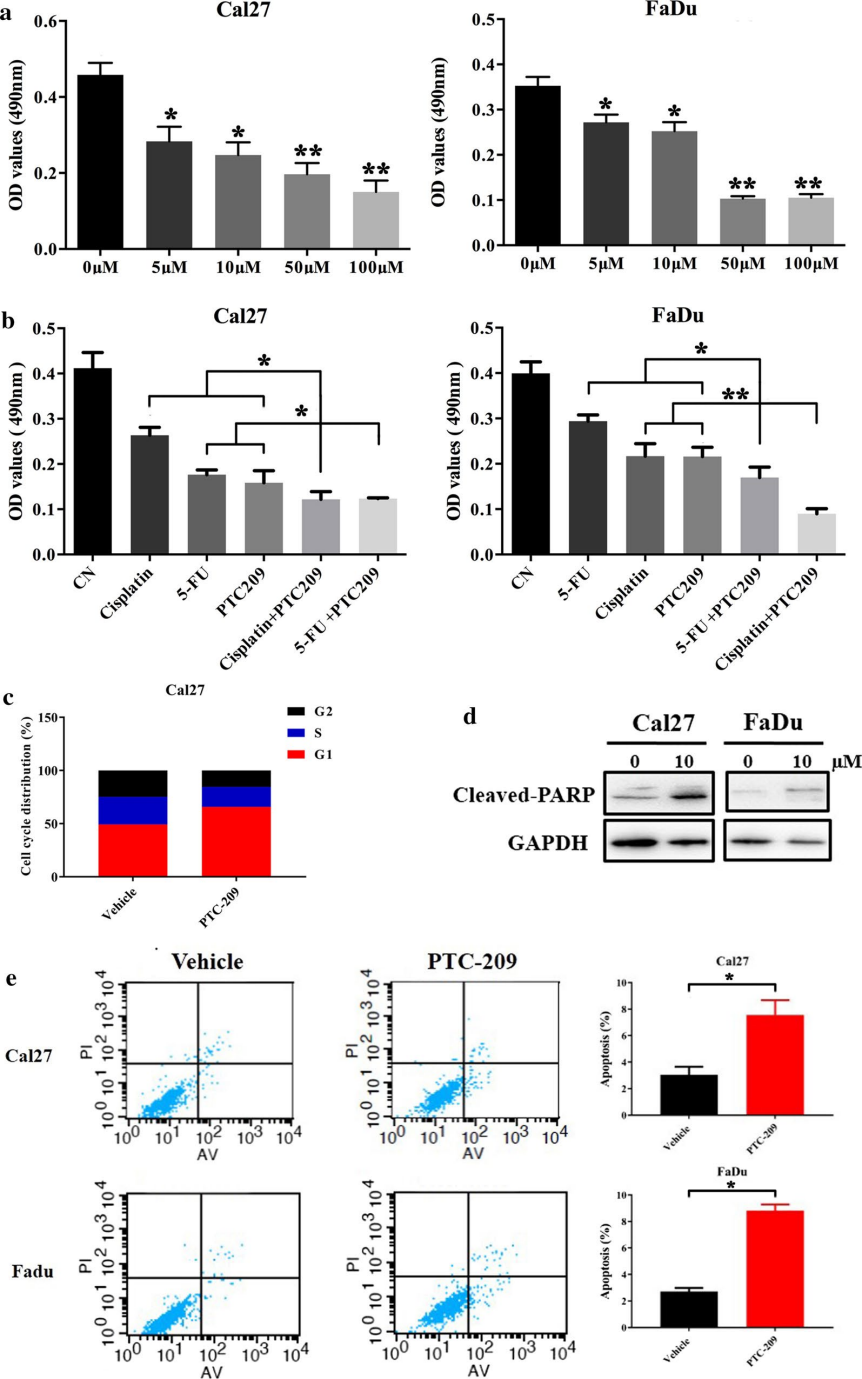
PTC-209 inhibited cell proliferation and induced cell cycle arrest, cell apoptosis and enhanced chemosensitivity in HNSCC cells. **a** Cell proliferation was remarkably suppressed in Cal27 and FaDu cells upon treatment with PTC-209 (10 μM) as measured by MTT assay. **b** Cell viability was more significantly impaired in cells treated with PTC-209 (10 μM) pus 5-FU (2.5 μg/ml) or cisplatin (2.5 μg/ml) than those treated with single agent. **c** Increased percentages of cells in G1 stage was observed in Cal27 treated with PTC-209 (10 μM) for 48 h. **d** Increased percentages of cell undergoing apoptosis (both early and late apoptosis) were detected in cells treated with PTC-209 (10 μM) as compared to control (vehicle-treated cells) as assayed by Annexin V-PI double staining. **e** The abundances of apoptosis marker cleave-PARP were concomitantly increased after PTC-209 exposure (10 μM) for 48 h. Representative images are shown. Data showed here are mean ± SD from three independent experiments, *p < 0.05, **p < 0.01, Student’s *t* test and ANOVA analyses

**Fig. 4:**
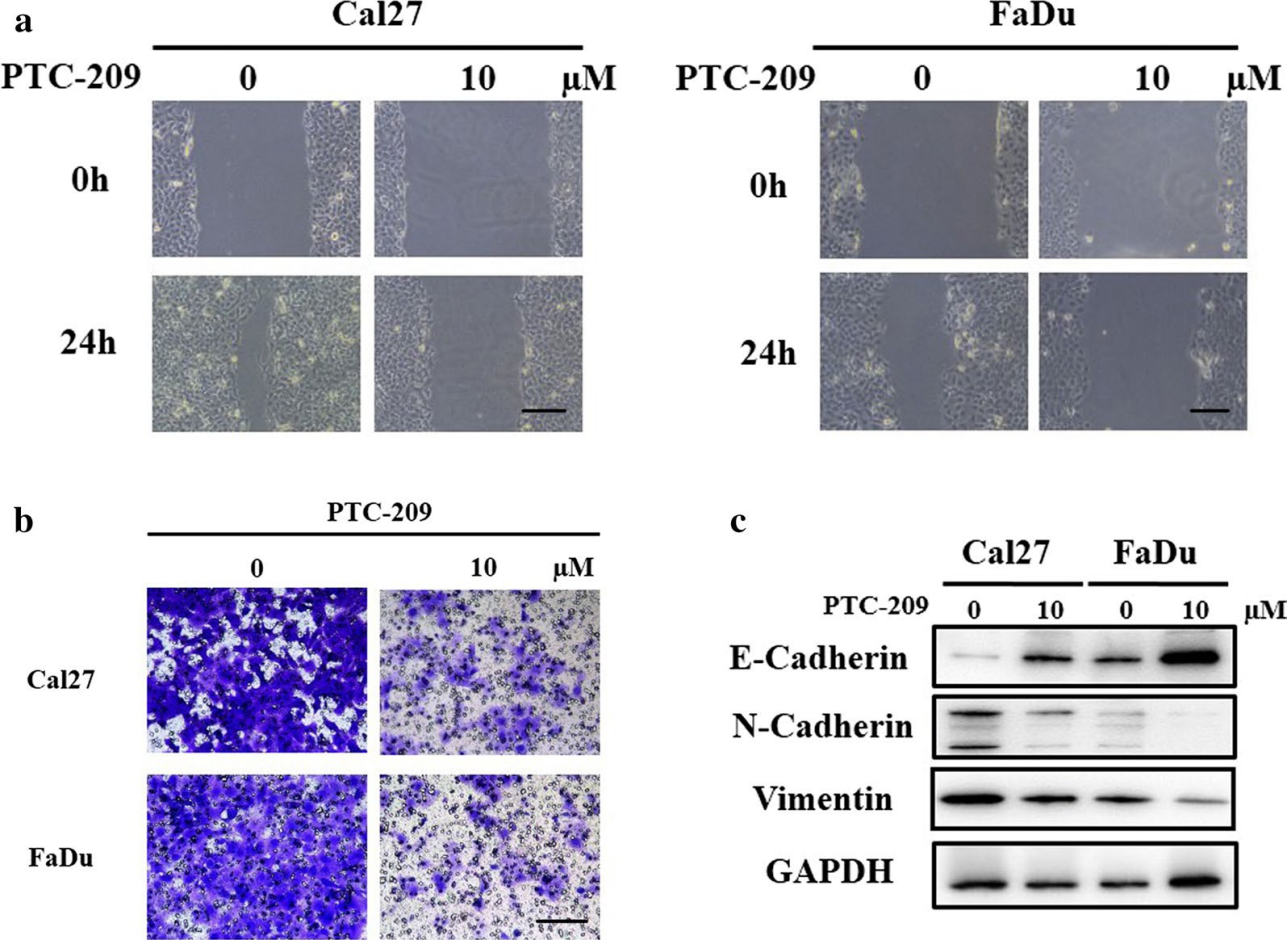
PTC-209 inhibited cell migration and invasion of HNSCC cells. **a** Cell migration was significantly reduced in cells treated with PTC-209 (10 μM, 24 h) as measured in wound healing assay. Scale bar: 200 μm. **b** Cell invasiveness was pronouncedly impaired in cells treated with PTC-209 (10 μM, 24 h) as measured in the transwell assay. Scale bar: 50 μm. **c** The amount of E-cadherin was increased and N-cadherin and vimentin was downregulated simultaneously in cells treated with PTC-209 (10 μM, 48 h). Representative images are shown. Data showed here are mean ± SD from three independent experiments

Having demonstrated the inhibitory effects of PTC-209 on Bmi1 expression in HNSCC, we next sought to determine the phenotypic changes upon PTC-209 in detail. As shown in Fig. 3a, when cells were treated with diverse concentrations of PTC-209, the cell proliferation was markedly suppressed as measured in MTT assay. In addition, when cells were treated with PTC-209 alone or together with 5-FU or cisplatin, the common therapeutic agents against HNSCC, our data revealed more obviously anti-proliferative effects of combinational treatments (PTC-209+5-FU, PTC-209+cisplatin) compared to single agent treatment (Fig. 3b), suggesting the potential synergic effects of PTC-209 and these agents. Moreover, cell cycle analysis revealed G1 arrest upon PTC-209 treatment in Cal27 and FaDu cells (Fig. 3c and data not shown). The proportions of cell undergoing apoptosis after PTC-209 exposure were significantly increased (Cal27 3.16% vs 7.81%; FaDu 2.84 vs 9.03%; *p* < 0.01) as evidenced by Annexin V-PI double staining and increased apoptosis marker cleaved-PARP (Fig. 3d, e). Given the proposed roles of Bmi1 during EMT and invasion in HNSCC [9, 13, 20], we next proceeded to determine whether PTC-209 affected the migratory and invasive potential of HNSCC cells. As anticipated, our data from wound healing and transwell assays showed that cell migration and invasion of both Cal27 and FaDu cells were significantly inhibited after PTC-209 exposure (10 μM, 48 h) as compared to vehicle-treated cells (Fig. 4a, b). In support of these findings, the invasion-associated EMT markers E-cadherin was upregulated while N-cadherin and Vimentin was concomitantly downregulated following PTC-209 addition (Fig. 4c).

### PTC-209 inhibited colony and tumorsphere formation and decreased the proportion of ALDH1^+^ subpopulation in HNSCC cells

**Fig. 5:**
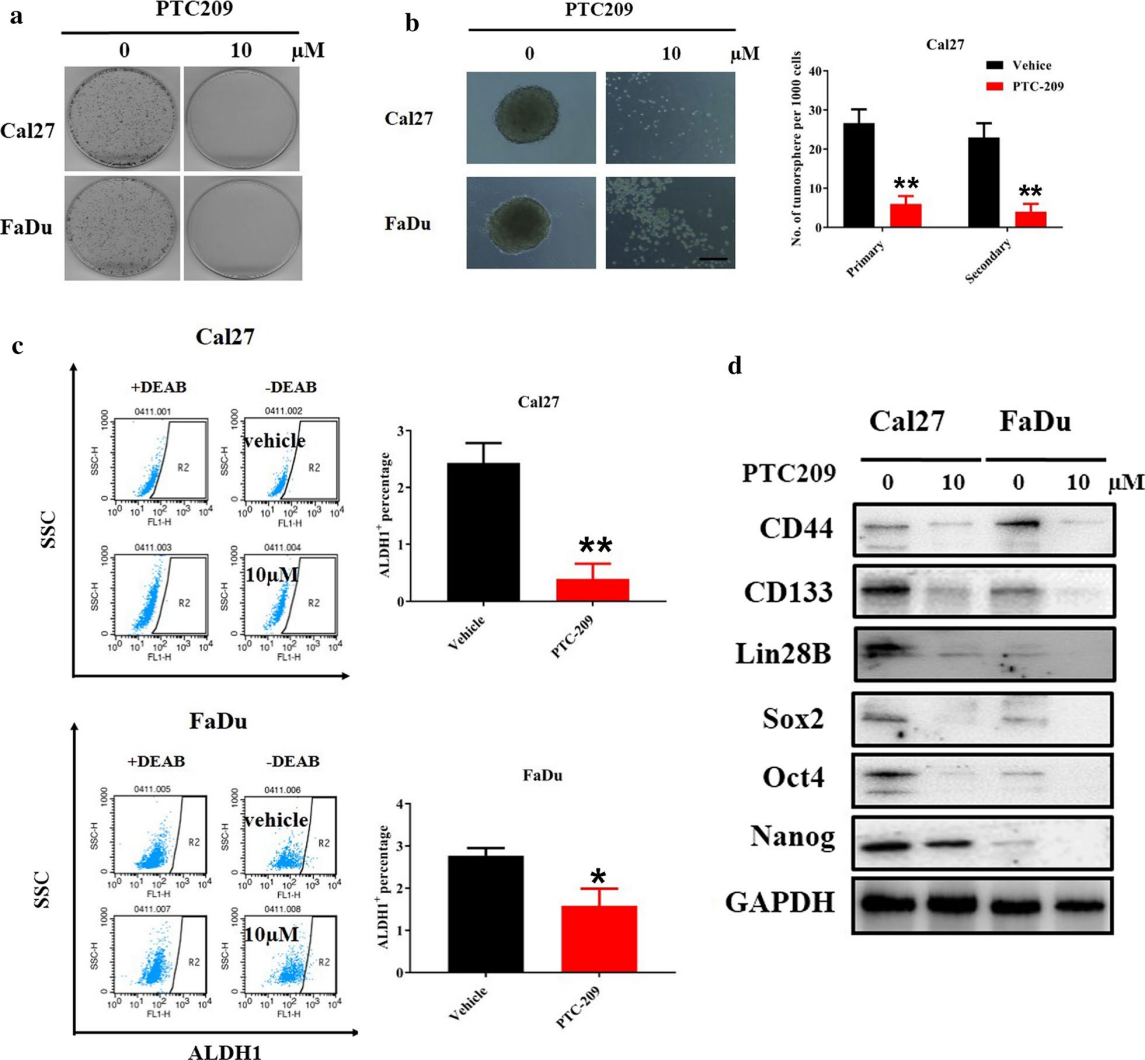
PTC-209 impaired colony and tumorsphere formation, and reduced the percentage of ALDH1^+^ and stemness markers expression in HNSCC cells. **a** The colony formation efficiency was pronouncedly reduced in cells pretreated with PTC-209 (10 μM) as compared to vehicle-treated cells. **b** The first and secondary rounds of tumorsphere formation was impaired in cells pretreated with PTC-209 (10 μM) as compared to vehicle-treated cells. Scale bar: 50 μm. The percentages of ALDH1^+^ subpopulation in Cal27 (**c**) and FaDu (**d**), which was enriched with putative CSCs, were significantly decreased after PTC-209 (10 μM ,48 h) as measured by ALDEFLUOR assay. **e** The expression of several CSCs markers was downregulated in PTC-209-treated cells as compared to vehicle-treated cells. Data showed here are mean ± SD from three independent experiments, *p < 0.05, **p < 0.01, Student’s *t* test and ANOVA analyses

Considering the critical roles of Bmi1 for CSCs self-renewal [13, 16, 22], we next sought to investigate the effects of PTC-209 on the CSCs-associated traits in HNSCC. Our data from colony formation assay revealed that PTC-209 pretreatment significantly reduced the numbers and size of colonies derived from both Cal27 and FaDu cells (Fig. 5a). Moreover, we utilized the tumorsphere formation assay, a surrogate test for CSCs stemness, to evaluate the effects of PTC-209 on the self-renewal properties of HNSCC cells. As expected, the number of tumorsphere formed in vitro was significantly decreased upon PTC-209 addition. Noticeably, secondary tumorsphere formation was also remarkably impaired in PTC-209-treated cells, thus suggesting that the self-renewal properties were compromised in HNSCC cells treated with PTC-209 (Fig. 5b). In parallel, the ALDH1^+^ subpopulation which was functionally enriched with putative CSCs [37] was also measured following PTC-209 treatment. As shown in Fig. 5c, d, the ratios of ALDH1^+^ cells were significantly reduced from 2.20 to 0.42% in Cal27 and from 2.85 to 1.32% in FaDu, respectively. Complemented with these findings, PTC-209 treatment also resulted in remarkable downregulation of several CSCs markers and modulators such as CD44, CD133, Sox2, Nanog et al. (Fig. 5e).

### Pharmacological inhibition of Bmi1 by PTC-209 impaired HNSCC overgrowth in vivo

**Fig. 6:**
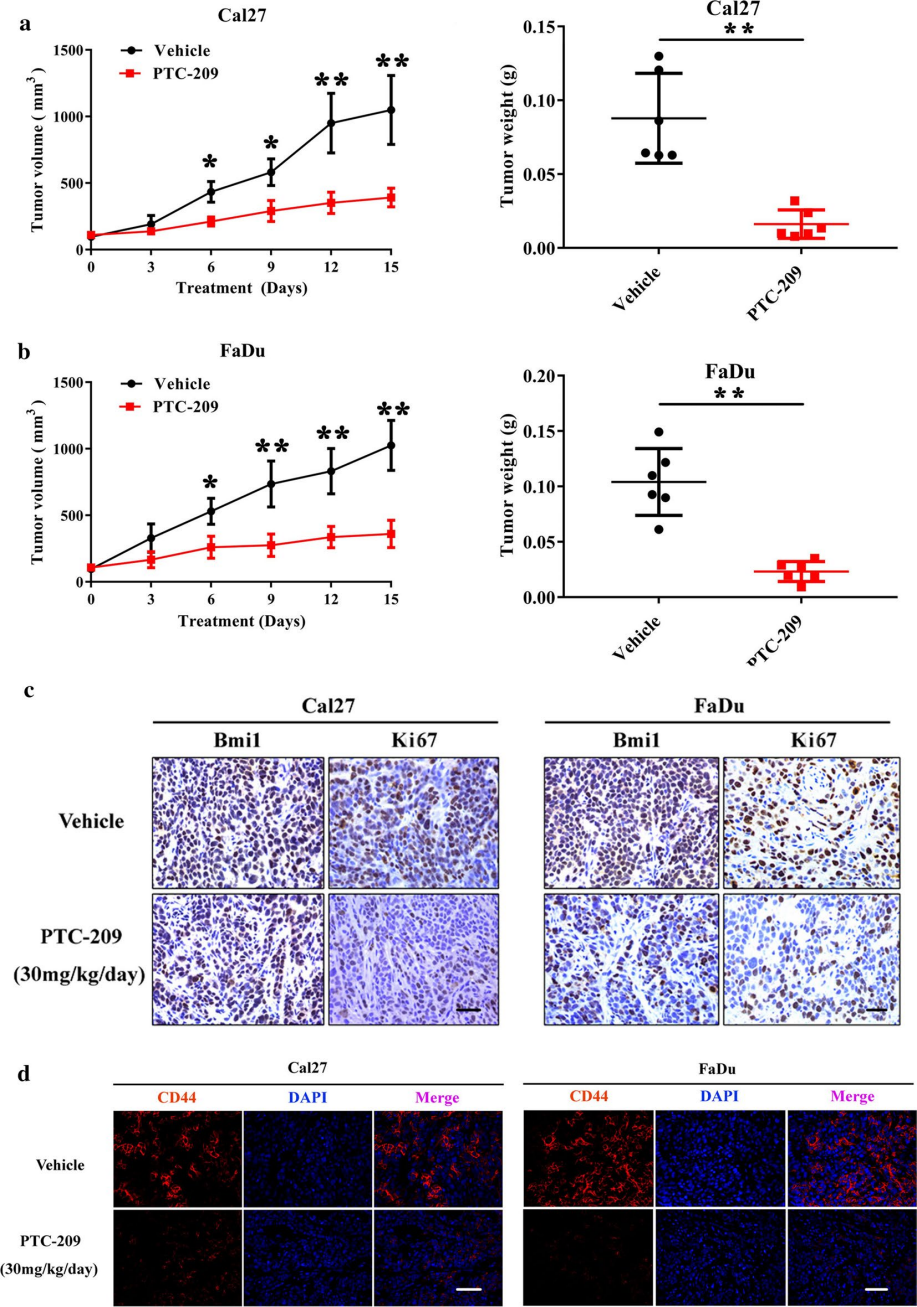
PTC-209 administration impairs tumor growth in a HNSCC xenograft model. **a** Volume changes in tumor masses harvested from PTC-209-treated (30 mg/kg, 15 days) animals and vehicle-treated animals were detected and compared. **b** Final weight of tumor masses harvested from PTC-209-treated animals and vehicle-treated animals was compared. **c** Immunohistochemical staining of Bmi1 and Ki67 in samples harvested from PTC-209-treated animals and vehicle-treated animals. Scale bar: 100 μm. **d** Immunoflurescent staining of CSCs surface marker CD44 in samples harvested from PTC-209-treated animals and vehicle-treated animals. Scale bar: 100 μm. Representative images are shown. *p < 0.05, **p < 0.01, Student’s *t* test

Having demonstrated the anticancer effects of PTC-209 in vitro, we then wanted to determine its therapeutic efficiency in vivo to further reinforce the translational potential of PTC-209 as a novel chemical against HNSCC. The HNSCC xenograft model was developed by subcutaneous transplantation of HNSCC cells in nude mice. After subcutaneous inoculation of Cal27 and FaDu cell for consecutive 3 weeks, tumor masses were established with approximate 100 mm^3^. The mice bearing tumors were randomly divided into groups for PTC-209 or vehicle treatment by subcutaneous injection. Tumor volume in recipient animals were monitored every 3 days by manual gauge with a caliber. During the whole experiment, all animals survived and had normal weight gains and undisturbed activities (data not shown), thus suggesting these treatments were well-tolerated in experimental animals. As shown in Fig. 6a, b, PTC-209 administration significantly retarded and impaired tumor overgrowth as compared with vehicle as measured by tumor volume and final weight of tumor masses, although tumor regression was not achieved under our experimental conditions. The samples derived from animals in each group were proceeded to routine H&E staining and immunohistochemical staining. As displayed in Fig. 6c, the number of Bmi1-positive cancer cells in PTC-209-treated samples was significantly less than those in vehicle-treated samples, therefore suggesting that Bmi1 was inhibited in vivo by PTC-209. Similarly, cancer cell proliferation was compromised in PTC-209-treated samples as evidenced by weaker and less Ki-67 staining (Fig. 6c and data not shown). Moreover, immunofluorescence staining of the putative HNSCC CSCs marker CD44 [38] revealed that the number of CD44-positive cells was much less in PTC-209-treated samples in relative to controls (Fig. 6d).

## Discussion

The polycomb complex mediates gene repression by modifying chromatin structure and is critically involved in various fundamental biological processes including stem cell homeostasis, cell differentiation and tumorigenesis [6]. In particular, Bmi1 has been increasingly recognized as a key bona fide oncogene driving tumorigenesis and a novel therapeutic target against human malignancy [18, 23]. In the present study, our data confirm that Bmi1 can be successfully inhibited by PTC-209 with high potency and specificity in HNSCC. Pharmacological targeting of Bmi1 resulted in impaired cell proliferation, migration and invasion in parallel to increased cellular apoptosis and reduced CSCs subpopulation. These findings highlight the Bmi1 is a feasible and viable therapeutic target against HNSCC with translational potentials.

Mounting evidence has established that Bmi1 is a key oncogene intricately linked to cell transformation, EMT and CSCs propagation in diverse cancer contexts including HNSCC [6, 9, 18]. Ectopic Bmi1 overexpression results in senescence bypass and immortalization of normal keratinocytes and malignant progression of precancerous lesions into cancers [24, 39, 40]. Our bioinformatics analyses by data mining and interrogation reveal that Bmi1 mRNA is aberrantly overexpressed in a fraction of, although not all, HNSCC samples. Previous studies including ours and others have indicated that Bmi1 protein is usually upregulated in HNSCC as compared to normal counterparts as determined by immunohistochemical staining in clinical samples [9, 15, 20, 24, 41]. Moreover, its expression status associated with aggressive clinicopathological features and outcomes, and served as an prognostic predictor for HNSCC [13, 15, 20, 24]. In contrast, Hayry V, etc. reported that Bmi1 protein expression didn’t correlate with any clinical and histopathological parameters and its negative expression served as adverse prognostic factor in patients with primary T1N0M0 tongue cancers [42]. Moreover, our findings using TCGA–HNSCC data also failed to reveal the differential expression of Bmi1 between cancer and normal epithelial and associations between Bmi1 expression and clinicopathological characteristics or prognosis. The reasons for this discrepancy remain unknown. We speculated that such conflicting findings might be partially due to diverse patient’s inclusion criteria, sample size, genetic background, immunohistochemical scoring and potential inconsistence between mRNA and protein abundance of a specific gene. Collectively, these findings indicate that aberrant Bmi1 overexpression is found in a subset of HNSCC and might serve as one of the key molecular events underlying HNSCC initiation and progression.

The well-established oncogenic roles of Bmi1 have spurred intensive efforts to uncover its potential as a therapeutic target for cancer. Previous studies have revealed that Bmi1 expression and activity were potently inhibited by HDACi presumably via transcriptional repression in multiple texts of malignancies [20, 43]. However, these HDACi compounds are not specific to Bmi1 and might induce unwanted side-effects as evidenced by our previous findings that Trichostatin A (TSA) potently inhibited Bmi1 but paradoxically induced EMT-like changes in HNSCC cells [20]. Recent pioneering studies have identified PTC-209 as a potent and specific chemical inhibitor of Bmi1 and revealed promising therapeutic efficiency of PTC-209 against cancer [12, 22]. Our data presented here are consistent with previous findings and further experimentally confirmed that PTC-209 is a potent inhibitor of Bmi1 in HNSCC as evidenced by remarkable downregulation of Bmi1 in vitro and in vivo. Furthermore, Bmi1 loss induced by PTC-209 resulted in similar phenotypic changes reminiscence of shRNA-mediated Bmi1 depletion in HNSCC cells, thus supporting Bmi1 as the target of PTC-209. Noticeably, we found that in addition to post-transcriptional regulation of Bmi1 expression by PTC-209 [22], the proteasomal degradation might be partially responsible for PTC-209-mediated Bmi1 downregulation in HNSCC as evidenced by the attenuation of Bmi1 loss by MG132 addition following PTC-209 treatment and increased ubiquitination of Bmi1 after PTC-209 exposure. This is conceivable and in agreement with previous findings Bmi1 protein is relatively unstable and has short half-life in cancer cells [36, 44, 45]. Of course, further investigations into mechanistic insights into PTC-209-induced Bmi1 loss in diverse cancers are still needed and warranted.

Accumulative evidence has revealed that Bmi1 has critical roles implicated in normal and malignant stem cell self-renewal and has been recognized as a functional marker as well as regulator for CSCs in malignancies including HNSCC [13, 17, 22, 46]. For example, CSCs properties in human colorectal cancer are highly dependent on Bmi1, as downregulation of Bmi1 inhibits their self-renewal and abrogates their tumorigenic potentials in vitro and in animal models [22]. Not surprisingly, our studies revealed that Bmi1 depletion mediated by PTC-209 significantly inhibited tumorsphere formation, reduced the percentage of CSCs subpopulation with concomitant downregulation of CSCs markers. In addition, PTC-209 also enhanced chemosensitivity and vulnerability of HNSCC cells to 5-FU and cisplatin. These findings largely phenocopied the Bmi1 knockdown [20], thus supporting that PTC-209 might selectively target the CSCs subpopulation via reducing Bmi1 in HNSCC. Indeed, when our manuscript was under preparation, Wang CY and his colleagues have reported that Bmi1 is a marker for HNSCC CSCs subpopulation through lineage tracing and also serves as a key driver mediating the metastasis and chemoresistance of HNSCC. Genetic ablation or pharmacological inhibition of Bmi1 markedly impaired 4NQO-induced tongue tumorigenesis, tumor overgrowth and cervical node metastasis [47]. Moreover, given conventional chemotherapy usually enriched putative CSCs [48, 49] and sometimes induced Bmi1 upregulation in HNSCC [50], combinational therapy consisting chemical agents and Bmi1 inhibitors might yield better outcomes than monotherapy for HNSCC. Collectively, these data strongly suggest that Bmi1 is a pivotal regulator for CSCs maintenance and can be therapeutically manipulated against HNSCC with promising benefits.

## Conclusion

Here our findings indicate that pharmacological depletion of Bmi1 in HNSCC by PTC-209 induces anti-neoplastic effects both in vitro and in vivo xenograft animal model. Disruption of Bmi1 by selective and potent chemical inhibitors might represent a novel therapeutic strategy against HNSCC, especially for patients with aberrant Bmi1 overexpression.

## Additional files

**Additional file 1: Figure S1.** Bmi1 mRNA expression in HNSCC samples derived from TCGA database. The original data of Bmi1 mRNA in HNSCC samples and normal epithelial from TCGA patient cohort were download and log2 transformed, and then statistically compared. ^#^ *p* > 0.05, Mann–Whitney U test.

**Additional file 2: Table S1.** Associations between Bmi1 mRNA expression and selected clinicopathological parameters in HNSCC.

**Additional file 3: Figure S2.** Global ubiquitinated histone 2A (uH2A), the hallmark of Bmi1-mediated repressive chromatin structures and transcriptional silencing, is significantly downregulated upon PTC-209 treatment (10 μM, 48 h). The representative images of western blot are shown.

## Abbreviations

HNSCC: head neck squamous cell carcinoma
HPV: human papillomavirus
PRC2: polycomb repressive complex 2
Bmi1: B lymphoma Mo-MLV insertion region 1 homolog
PRC1: Polycomb Repressive Complex 1
EMT: epithelial–mesenchymal transition
CSCs: cancer stem cells
HDACi: inhibitors of histone deacetylases
EGF: epidermal growth factor
bFGF: basic fibroblast growth factor

## References

[1] Siegel RL, Miller KD, Jemal A (2017). Cancer statistics, 2017. CA Cancer J Clin.

[2] Miller KD, Siegel RL, Lin CC, Mariotto AB, Kramer JL, Rowland JH, Stein KD, Alteri R, Jemal A (2016). Cancer treatment and survivorship statistics, 2016. CA Cancer J Clin.

[3] Cancer Genome Atlas N (2015). Comprehensive genomic characterization of head and neck squamous cell carcinomas. Nature.

[4] Leemans CR, Braakhuis BJ, Brakenhoff RH (2011). The molecular biology of head and neck cancer. Nat Rev Cancer.

[5] Haddad RI, Shin DM (2008). Recent advances in head and neck cancer. N Engl J Med.

[6] Sauvageau M, Sauvageau G (2010). Polycomb group proteins: multi-faceted regulators of somatic stem cells and cancer. Cell Stem Cell.

[7] Jacobs JJ, Kieboom K, Marino S, DePinho RA, van Lohuizen M (1999). The oncogene and Polycomb-group gene bmi-1 regulates cell proliferation and senescence through the ink4a locus. Nature.

[8] Lukacs RU, Memarzadeh S, Wu H, Witte ON (2010). Bmi-1 is a crucial regulator of prostate stem cell self-renewal and malignant transformation. Cell Stem Cell.

[9] Yang MH, Hsu DS, Wang HW, Wang HJ, Lan HY, Yang WH, Huang CH, Kao SY, Tzeng CH, Tai SK (2010). Bmi1 is essential in Twist1-induced epithelial–mesenchymal transition. Nat Cell Biol.

[10] Chiba T, Miyagi S, Saraya A, Aoki R, Seki A, Morita Y, Yonemitsu Y, Yokosuka O, Taniguchi H, Nakauchi H (2008). The polycomb gene product BMI1 contributes to the maintenance of tumor-initiating side population cells in hepatocellular carcinoma. Cancer Res.

[11] Elkhadragy L, Chen M, Miller K, Yang MH, Long W (2017). A regulatory BMI1/let-7i/ERK3 pathway controls the motility of head and neck cancer cells. Mol Oncol.

[12] Yong KJ, Basseres DS, Welner RS, Zhang WC, Yang H, Yan B, Alberich-Jorda M, Zhang J, de Figueiredo-Pontes LL, Battelli C (2016). Targeted BMI1 inhibition impairs tumor growth in lung adenocarcinomas with low CEBPalpha expression. Sci Transl Med..

[13] He Q, Liu Z, Zhao T, Zhao L, Zhou X, Wang A (2015). Bmi1 drives stem-like properties and is associated with migration, invasion, and poor prognosis in tongue squamous cell carcinoma. Int J Biol Sci.

[14] Tu Y, Gao X, Li G, Fu H, Cui D, Liu H, Jin W, Zhang Y (2013). MicroRNA-218 inhibits glioma invasion, migration, proliferation, and cancer stem-like cell self-renewal by targeting the polycomb group gene Bmi1. Cancer Res.

[15] Chou CH, Yang NK, Liu TY, Tai SK, Hsu DS, Chen YW, Chen YJ, Chang CC, Tzeng CH, Yang MH (2013). Chromosome instability modulated by BMI1-AURKA signaling drives progression in head and neck cancer. Cancer Res.

[16] Gargiulo G, Cesaroni M, Serresi M, de Vries N, Hulsman D, Bruggeman SW, Lancini C, van Lohuizen M (2013). In vivo RNAi screen for BMI1 targets identifies TGF-beta/BMP-ER stress pathways as key regulators of neural- and malignant glioma-stem cell homeostasis. Cancer Cell.

[17] Smith LL, Yeung J, Zeisig BB, Popov N, Huijbers I, Barnes J, Wilson AJ, Taskesen E, Delwel R, Gil J (2011). Functional crosstalk between Bmi1 and MLL/Hoxa9 axis in establishment of normal hematopoietic and leukemic stem cells. Cell Stem Cell.

[18] Siddique HR, Saleem M (2012). Role of BMI1, a stem cell factor, in cancer recurrence and chemoresistance: preclinical and clinical evidences. Stem Cells.

[19] Ferretti R, Bhutkar A, McNamara MC, Lees JA (2016). BMI1 induces an invasive signature in melanoma that promotes metastasis and chemoresistance. Genes Dev.

[20] Li Z, Wang Y, Yuan C, Zhu Y, Qiu J, Zhang W, Qi B, Wu H, Ye J, Jiang H (2014). Oncogenic roles of Bmi1 and its therapeutic inhibition by histone deacetylase inhibitor in tongue cancer. Lab Invest.

[21] Nishida Y, Maeda A, Chachad D, Ishizawa J, Qiu YH, Kornblau SM, Kimura S, Andreeff M, Kojima K (2015). Preclinical activity of the novel B-cell-specific Moloney murine leukemia virus integration site 1 inhibitor PTC-209 in acute myeloid leukemia: implications for leukemia therapy. Cancer Sci.

[22] Kreso A, van Galen P, Pedley NM, Lima-Fernandes E, Frelin C, Davis T, Cao L, Baiazitov R, Du W, Sydorenko N (2014). Self-renewal as a therapeutic target in human colorectal cancer. Nat Med.

[23] Cao L, Bombard J, Cintron K, Sheedy J, Weetall ML, Davis TW (2011). BMI1 as a novel target for drug discovery in cancer. J Cell Biochem.

[24] Kang MK, Kim RH, Kim SJ, Yip FK, Shin KH, Dimri GP, Christensen R, Han T, Park NH (2007). Elevated Bmi-1 expression is associated with dysplastic cell transformation during oral carcinogenesis and is required for cancer cell replication and survival. Br J Cancer.

[25] Yamazaki H, Mori T, Yazawa M, Maeshima AM, Matsumoto F, Yoshimoto S, Ota Y, Kaneko A, Tsuda H, Kanai Y (2013). Stem cell self-renewal factors Bmi1 and HMGA2 in head and neck squamous cell carcinoma: clues for diagnosis. Lab Invest.

[26] Wei Z, Wang Y, Li Z, Yuan C, Zhang W, Wang D, Ye J, Jiang H, Wu Y, Cheng J (2013). Overexpression of Hippo pathway effector TAZ in tongue squamous cell carcinoma: correlation with clinicopathological features and patients’ prognosis. J Oral Pathol Med.

[27] Liu LK, Jiang XY, Zhou XX, Wang DM, Song XL, Jiang HB (2010). Upregulation of vimentin and aberrant expression of E-cadherin/beta-catenin complex in oral squamous cell carcinomas: correlation with the clinicopathological features and patient outcome. Mod Pathol.

[28] Shin G, Kang TW, Yang S, Baek SJ, Jeong YS, Kim SY (2011). GENT: gene expression database of normal and tumor tissues. Cancer Inform.

[29] Cerami E, Gao J, Dogrusoz U, Gross BE, Sumer SO, Aksoy BA, Jacobsen A, Byrne CJ, Heuer ML, Larsson E (2012). The cBio cancer genomics portal: an open platform for exploring multidimensional cancer genomics data. Cancer Discov.

[30] Rhodes DR, Kalyana-Sundaram S, Mahavisno V, Varambally R, Yu J, Briggs BB, Barrette TR, Anstet MJ, Kincead-Beal C, Kulkarni P (2007). Oncomine 3.0: genes, pathways, and networks in a collection of 18,000 cancer gene expression profiles. Neoplasia.

[31] Toruner GA, Ulger C, Alkan M, Galante AT, Rinaggio J, Wilk R, Tian B, Soteropoulos P, Hameed MR, Schwalb MN (2004). Association between gene expression profile and tumor invasion in oral squamous cell carcinoma. Cancer Genet Cytogenet.

[32] Ginos MA, Page GP, Michalowicz BS, Patel KJ, Volker SE, Pambuccian SE, Ondrey FG, Adams GL, Gaffney PM (2004). Identification of a gene expression signature associated with recurrent disease in squamous cell carcinoma of the head and neck. Cancer Res.

[33] Cromer A, Carles A, Millon R, Ganguli G, Chalmel F, Lemaire F, Young J, Dembele D, Thibault C, Muller D (2004). Identification of genes associated with tumorigenesis and metastatic potential of hypopharyngeal cancer by microarray analysis. Oncogene.

[34] Kuriakose MA, Chen WT, He ZM, Sikora AG, Zhang P, Zhang ZY, Qiu WL, Hsu DF, McMunn-Coffran C, Brown SM (2004). Selection and validation of differentially expressed genes in head and neck cancer. Cell Mol Life Sci.

[35] Peng CH, Liao CT, Peng SC, Chen YJ, Cheng AJ, Juang JL, Tsai CY, Chen TC, Chuang YJ, Tang CY (2011). A novel molecular signature identified by systems genetics approach predicts prognosis in oral squamous cell carcinoma. PLoS ONE.

[36] Yadav AK, Sahasrabuddhe AA, Dimri M, Bommi PV, Sainger R, Dimri GP (2010). Deletion analysis of BMI1 oncoprotein identifies its negative regulatory domain. Mol Cancer.

[37] Chen YC, Chen YW, Hsu HS, Tseng LM, Huang PI, Lu KH, Chen DT, Tai LK, Yung MC, Chang SC (2009). Aldehyde dehydrogenase 1 is a putative marker for cancer stem cells in head and neck squamous cancer. Biochem Biophys Res Commun.

[38] Prince ME, Sivanandan R, Kaczorowski A, Wolf GT, Kaplan MJ, Dalerba P, Weissman IL, Clarke MF, Ailles LE (2007). Identification of a subpopulation of cells with cancer stem cell properties in head and neck squamous cell carcinoma. Proc Natl Acad Sci USA.

[39] Liu W, Feng JQ, Shen XM, Wang HY, Liu Y, Zhou ZT (2012). Two stem cell markers, ATP-binding cassette, G2 subfamily (ABCG2) and BMI-1, predict the transformation of oral leukoplakia to cancer: a long-term follow-up study. Cancer.

[40] Qiao B, Chen Z, Hu F, Tao Q, Lam AK (2013). BMI-1 activation is crucial in hTERT-induced epithelial–mesenchymal transition of oral epithelial cells. Exp Mol Pathol.

[41] Huber GF, Albinger-Hegyi A, Soltermann A, Roessle M, Graf N, Haerle SK, Holzmann D, Moch H, Hegyi I (2011). Expression patterns of Bmi-1 and p16 significantly correlate with overall, disease-specific, and recurrence-free survival in oropharyngeal squamous cell carcinoma. Cancer.

[42] Hayry V, Makinen LK, Atula T, Sariola H, Makitie A, Leivo I, Keski-Santti H, Lundin J, Haglund C, Hagstrom J (2010). Bmi-1 expression predicts prognosis in squamous cell carcinoma of the tongue. Br J Cancer.

[43] Bommi PV, Dimri M, Sahasrabuddhe AA, Khandekar J, Dimri GP (2010). The polycomb group protein BMI1 is a transcriptional target of HDAC inhibitors. Cell Cycle.

[44] Banerjee Mustafi S, Chakraborty PK, Dwivedi SK, Ding K, Moxley KM, Mukherjee P, Bhattacharya R (2017). BMI1, a new target of CK2alpha. Mol Cancer.

[45] Sahasrabuddhe AA, Dimri M, Bommi PV, Dimri GP (2011). betaTrCP regulates BMI1 protein turnover via ubiquitination and degradation. Cell Cycle.

[46] Tanaka T, Komai Y, Tokuyama Y, Yanai H, Ohe S, Okazaki K, Ueno H (2013). Identification of stem cells that maintain and regenerate lingual keratinized epithelial cells. Nat Cell Biol.

[47] Chen D, Wu M, Li Y, Chang I, Yuan Q, Ekimyan-Salvo M, Deng P, Yu B, Yu Y, Dong J (2017). Targeting BMI1+ Cancer stem cells overcomes chemoresistance and inhibits metastases in squamous cell carcinoma. Cell Stem Cell..

[48] Liu YP, Yang CJ, Huang MS, Yeh CT, Wu AT, Lee YC, Lai TC, Lee CH, Hsiao YW, Lu J (2013). Cisplatin selects for multidrug-resistant CD133+ cells in lung adenocarcinoma by activating Notch signaling. Cancer Res.

[49] Samanta D, Gilkes DM, Chaturvedi P, Xiang L, Semenza GL (2014). Hypoxia-inducible factors are required for chemotherapy resistance of breast cancer stem cells. Proc Natl Acad Sci USA.

[50] Nor C, Zhang Z, Warner KA, Bernardi L, Visioli F, Helman JI, Roesler R, Nor JE (2014). Cisplatin induces Bmi-1 and enhances the stem cell fraction in head and neck cancer. Neoplasia.

