# Supplementary figures and images for: Pharmacological inhibition of Bmi1 by PTC-209 impaired tumor growth in head neck squamous cell carcinoma
Source: Cancer Cell Int. 2017 Nov 21;17:107. doi: 10.1186/s12935-017-0481-z (PMC5697105; doi:10.1186/s12935-017-0481-z)

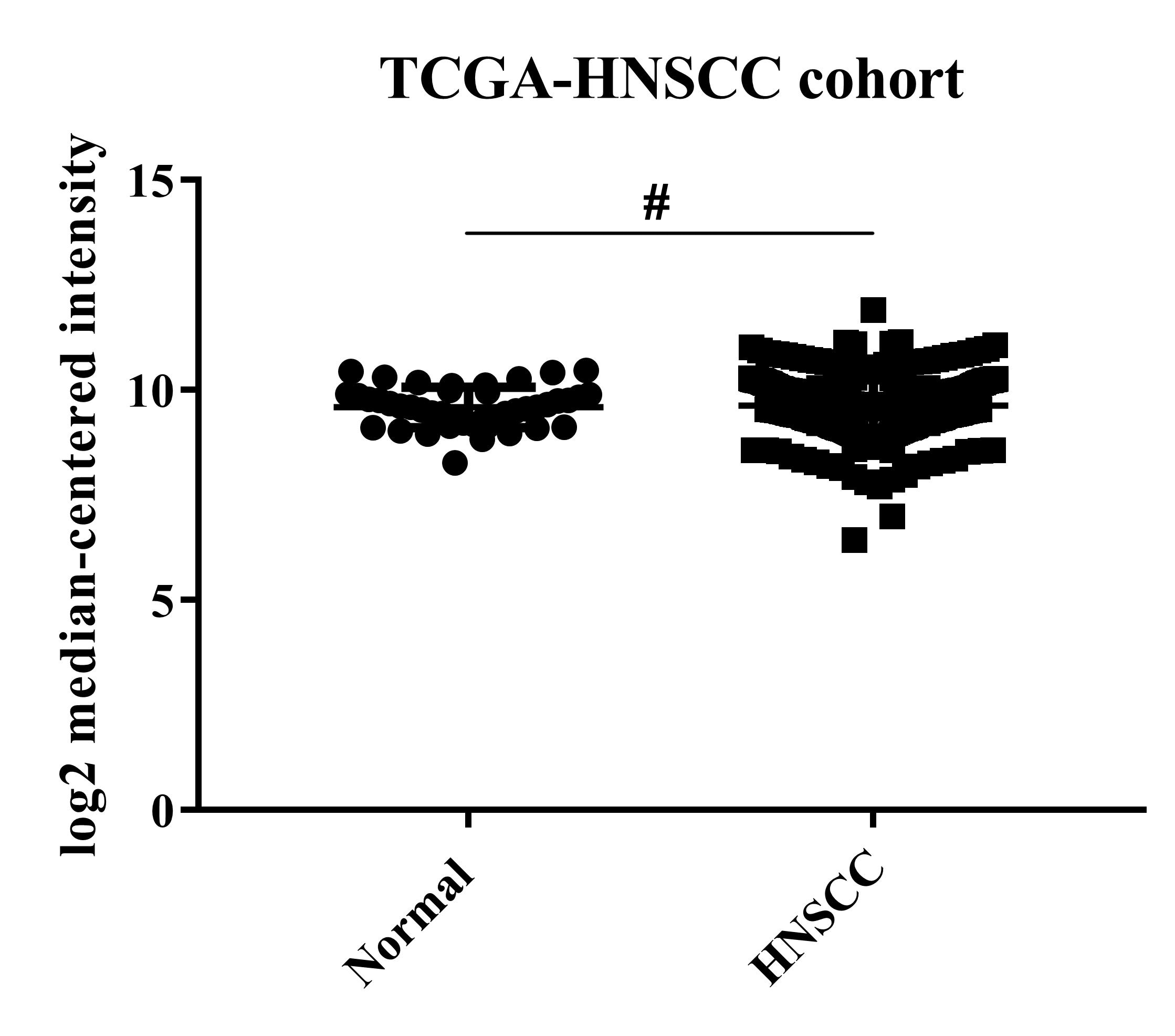

Supplement: Supplementary file 1 — Additional file 1: Figure S1. Bmi1 mRNA expression in HNSCC samples derived from TCGA database. The original data of Bmi1 mRNA in HNSCC samples and normal epithelial from TCGA patient cohort were download and log2 transformed, and then statistically compared. # p > 0.05, Mann–Whitney U test. [file 12935_2017_481_MOESM1_ESM.jpg]

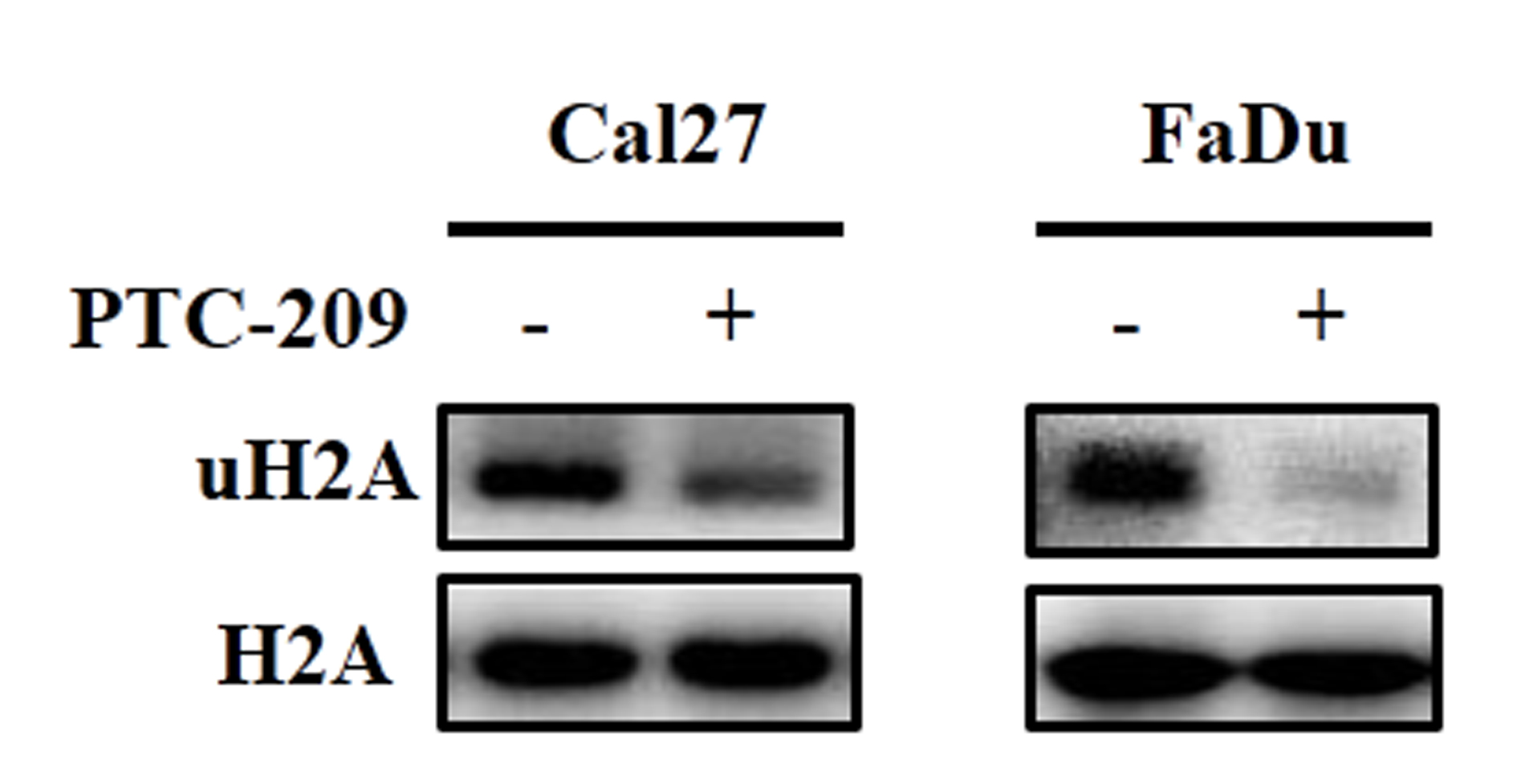

Supplement: Supplementary file 3 — Additional file 3: Figure S2. Global ubiquitinated histone 2A (uH2A), the hallmark of Bmi1-mediated repressive chromatin structures and transcriptional silencing, is significantly downregulated upon PTC-209 treatment (10 μM, 48 h). The representative images of western blot are shown. [file 12935_2017_481_MOESM3_ESM.jpg]
