# Supplementary material for: Pharmacological inhibition of Bmi1 by PTC-209 impaired tumor growth in head neck squamous cell carcinoma
Source: Cancer Cell Int. 2017 Nov 21;17:107. doi: 10.1186/s12935-017-0481-z (PMC5697105; doi:10.1186/s12935-017-0481-z)
Supplement: Supplementary file 2 — Additional file 2: Table S1. Associations between Bmi1 mRNA expression and selected clinicopathological parameters in HNSCC. [file 12935_2017_481_MOESM2_ESM.docx]

**Additional Table S1 Associations between Bmi1 mRNA expression and selected clinicopathological parameters in HNSCC**

| **Clinicopathological parameters** | **Cases** | **Bmi1** | | ***p-*values** |
| --- | --- | --- | --- | --- |
|  |  | **low** | **High** |  |
| **Gender** | 432* | 216 | 216 |  |
| Male | 313 | 66 | 53 | 0.1961 |
| Female | 119 | 150 | 163 |  |
| **Age** |  |  |  |  |
| ≤60 | 191 | 88 | 103 | 0.1749 |
| ＞60 | 241 | 128 | 113 |  |
| **Pathological stage** |  |  |  |  |
| Ⅰ  II | 26  71 | 16  35 | 10  36 | 0.5881 |
|  |  |  |  |  |
| Ⅲ | 74 | 39 | 35 |  |
| Ⅳ | 261 | 126 | 135 |  |
| **Clinical stage** |  |  |  |  |
| Ⅰ | 20 | 11 | 9 | 0.7999 |
| II | 87 | 45 | 42 |  |
| Ⅲ | 91 | 48 | 43 |  |
| Ⅳ | 234 | 112 | 122 |  |

* A total number of 432 patients with complete data concerning patient demographic, clinical and follow-up was retrieved from the original TCGA HNSCC database. The median value of Bmi1 mRNA was used as cutoff between low and high expression.
